# Liberal vs. restricted opioid prescribing following midurethral sling dataset

**DOI:** 10.1016/j.dib.2023.109144

**Published:** 2023-04-12

**Authors:** Brianne M. Morgan, Jaime B. Long, Sarah S. Boyd, Matthew F. Davies, Allen R. Kunselman, Christy M. Stetter, Michael H. Andreae

**Affiliations:** a Department of Obstetrics and Gynecology, Penn State Health Milton S. Hershey Medical Center, 500 University Drive, Hershey, PA 17033, USA; b Department of Public Health Sciences, Penn State Health Milton S. Hershey Medical Center, 500 University Drive, Hershey, PA 17033, USA; c Department of Anesthesiology, University of Utah Hospital, 50 N. Medical Drive, Salt Lake City, UT 84132, USA

**Keywords:** Opioid use, Opioid stewardship, Midurethral sling, Opioid prescribing, Randomized clinical trial

## Abstract

Postoperative opioid prescribing has historically lacked information critical to balancing the pain control needs of the individual patient with our professional responsibility to judiciously prescribe these high-risk medications. This data evaluates pain control, satisfaction with pain control, and opioid utilization among patients undergoing isolated mid-urethral sling (MUS) randomized to one of two different opioid prescribing regimens. This study was registered on clinicaltrials.gov (NCT04277975). Women undergoing isolated MUS by a Female Pelvic Medicine and Reconstructive Surgery physician at a Penn State Health hospital from June 1, 2020 to November 22, 2021 were offered enrollment into this prospective, randomized, open-label, non-inferiority clinical trial. Participants gave informed consent and were enrolled by a member of the study team. Allocation was concealed to patient and study personnel until randomization on the day of surgery. Preoperatively, all participants completed baseline demographic and pain surveys including CSI-9, PCS, and Likert pain score (scale 0-10). Participants were randomized to either receive a standard prescription of ten 5 mg tablets oxycodone provided preoperatively (standard) or opioid prescription provided only upon patient request postoperatively (restricted). Randomization was performed by the study team surgeon using the REDCap randomization module on the day of surgery.

Following MUS, subjects completed a daily diary for 1 week, i.e., postoperative day (POD) 0 through 7. Within the dairy, subjects provided the following information: average daily pain score, opioid use and amount of opioid utilized, other forms of pain management, satisfaction with pain control, perception of the amount of opioid prescribed, and need for pain management hospital/clinic visits. The online Prescription Drug Monitoring Program (PDMP) was queried for all patients to determine if prescriptions for opioids were filled during the postoperative period. The primary outcome was average postoperative day 1 pain score and an a priori determined margin of non-inferiority was set at 2 points. Secondary outcomes included whether subject filled an opioid prescription (indicated by the online PDMP), opioid use (yes/no), satisfaction with pain control (on a scale of 1= “much worse” to 5= “much better” than expected), and how subjects felt about the amount of opioid prescribed (on a scale of 1=“prescribed far more” to 3=“prescribed the right amount” to 5=“prescribed far less” opioid than needed). 82 participants underwent isolated MUS placement and met inclusion criteria; 40 were randomized to the standard arm and 42 to the restricted group. Within this manuscript, we detail the data obtained from this randomized clinical trial and the methods utilized.

**Specifications Table**

**Table:** 

Subject	Gynaecology
Specific subject area	Urogynecology: Midurethral Slings
Type of data	Figure dataset
How the data were acquired	Preoperative data was collected through the Electronic Medical Record (Cerner), and surveys including the 9-question Central Sensitization Index (CSI-9), 13 item Pain Catastrophizing Scale (PCS), and Likert Pain Scale. This data was collected from the patient either at the pre-operative appointment or the day of surgery in the pre-operative area. Randomization was performed on the day of surgery using REDCap electronic data capture tools hosted at Penn State Health Milton S. Hershey Medical Center and Penn State College of Medicine. NIH funding for RedCap, which implies compliance with NIH public access policy.^3^ A daily survey was completed by subjects on day 0-7 postoperatively either in REDCap database or in paper diary. The online Prescription Drug Monitoring Program (PDMP) was queried for all patients to determine if prescriptions for opioids were filled during the postoperative period. Analyses were performed using SAS Software, version 9.4^4^.
Data format	Raw
Description of data collection	Women undergoing isolated MUS by a Female Pelvic Medicine and Reconstructive Surgery physician at a Penn State Health hospital from June 1, 2020 to November 22, 2021 were eligible. Patients who were pregnant, nursing, cognitively impaired, currently using daily opioid or with opioid use disorder, allergic to oxycodone or acetaminophen/NSAID, or unable to speak or read English were excluded.
Data source location	• Institution: Penn State Health Milton S. Hershey Medical Center • City/Town/Region: Hershey, PA • Country: USA • Latitude and longitude for collected samples/data: 40.2640° N, 76.6766° W
Data accessibility	Repository name: Mendeley Data Data identification number: DOI:10.17632/rbcxdzf5c8.2 Direct URL to data: https://data.mendeley.com/datasets/rbcxdzf5c8/2[2].
Related research article	Long JB, Morgan BM, Boyd SS, Davies MF, Kunselman AR, Stetter CM, Andreae MH. A randomized trial of standard vs restricted opioid prescribing following midurethral sling. Am J Obstet Gynecol. 2022 Aug;227(2):313.e1-313.e9. doi:10.1016/j.ajog.2022.05.010. Epub 2022 May 10. PMID: 35550371 [1].

## Value of the Data

• This data is useful as it provides quantification on the amount of pain experienced and opioids used after mid-urethral sling. There are descriptive demographic data, psychometric survey data, and other postoperative subjective and objective postoperative outcome data.
• This benefits individuals who prescribe opioids and perform female pelvic and reconstructive surgery procedures. Additionally, this data will be useful to researchers interested in postoperative pain experiences, opioid use, and pain phenotyping.
• This data can be applied to hone the predictive ability of clinical, surgical, and psychometric factors related to postoperative pain and opioid needs.

## 1 Objective

This manuscript was created in order to expand upon postoperative pain management and risk factors for increased postoperative pain management needs. The dataset adds value to the original research article by sharing all methodology and findings that were not expanded upon in the original article, increasing ease for replication. All data accumulated from subjects rather than only data selected by research team is included. The sample size and power in this study was not large enough to identify risk factors for pain management. This dataset could be used by researchers who share similar aims to identify risk factors from a larger sample size.

## 2 Data Description

### 2.1 Raw Data

The raw data in the data repository includes every piece of information acquired from each subject. The data was exported from REDCap to an Excel document. Each subject corresponds to a “record ID”. Every record ID is listed in 11 consecutive rows to include baseline information, postoperative day 0 through 7 data entries, end of study data, and adverse events. An additional tab includes the data dictionary which describes the keys used for each response. Descriptive data including information on ethnicity, race, tobacco use, alcohol use, marital status, provider, and insurance all coordinated with a number, as seen in the data dictionary. Other data collected that coincided with the exact integer included age, BMI, ASA score, parity, and baseline pain score. Score of one for yes or zero for no was given if the patient did or did not have depression, anxiety, panic attacks, fibromyalgia, restless leg syndrome, chronic fatigue, temporomandibular joint disorder, migraines, chronic pelvic pain, irritable bowel syndrome, interstitial cystitis, multiple chemical sensitivities, or pelvic floor tension myalgia. Responses to each question on the PCS survey are included. The answers to each question corresponded with a zero (not at all), one (to a slight degree), two (to a moderate degree), three (to a great degree) or four (all the time). Responses to each question on the CSI nine questionnaire are included. The answers to each question corresponded to a zero (never), one (rarely), two (sometimes), three (often), or four (always). All baseline data described above in included in the “baseline arm” for each record ID. The daily post-operative diary data set was completed a total of eight times from post-operative day zero to seven. Each day included the following data. Average daily pain score zero to ten, number of opioid tablets used, Opioid morphine milligram equivalents used, other pain medication is used (one for ibuprofen, two for acetaminophen, three for other, four for none), other methods used for pain (one for heat, two for ice, three for pressure, four for other, five for none), satisfaction with pain control (1 for much worse than expected, two for worse than expected, three for same as expected, four for better than expected, five for way better than expected), and the feeling of amount of opioid prescribed (one for much more than needed, two for more than needed, three for the right amount, four for less than needed, five for much less than needed). The data from each postoperative day is logged for each record ID (eg. Pod0 arm, pod1 arm, etc.). Information from the PACU on day of surgery was collected including the amount of opioid used and pain score prior to discharge. It was recorded if the patient had filled an opioid prescription (one for none prescribed, two for prescribed but not filled by patient, three for filled by patient) and if an additional prescription was requested. This information is presented in the data set for each record ID as “end of study arm”. Adverse events were logged and categorized by systems affected and information was logged regarding the date, seriousness, if problem is ongoing, intensity, outcome, and if this was expected. This is logged as the last data row for each record ID as “end of study arm/ adverse event log”.

**Fig. 1:**
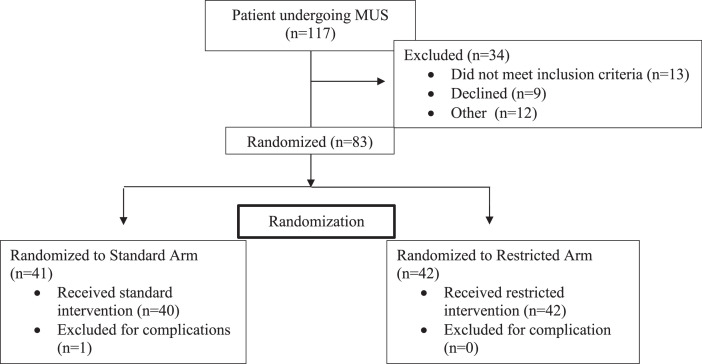
Participant diagram: quantity of subjects involved in enrollment, randomization, follow up, and analysis. 117 subjects were assessed for eligibility. A total of 34 were not included in randomization (13 did not meet inclusion criteria, 9 declined to participate, and 12 were missed at appointments). A total of 83 subjects were randomized the day of surgery; 1 patient was later excluded for complications incurred during surgery and inability to place sling. The final cohort for analysis included 82 patients; 40 standard arm and 42 restricted arm. No subjects were lost to follow up or excluded from analysis.

## 3 Experimental Design, Materials and Methods

Women 18 years and older undergoing isolated MUS by a Female Pelvic Medicine and Reconstructive Surgery physician at a Penn State Health hospital from June 1, 2020 to November 22, 2021 were offered enrollment into this prospective, randomized, open-label, non-inferiority clinical trial. Patients who were pregnant, breastfeeding, cognitively impaired, currently using daily opioid or with opioid use disorder, prisoners, allergic to oxycodone or acetaminophen/NSAID, or unable to speak or read English were excluded. Participants gave informed consent and were enrolled by a member of the study team at their preoperative appointments or in the pre-operative area the morning of their scheduled surgery. Allocation was concealed to patient and study personnel until randomization on the day of surgery. Preoperatively, all participants completed baseline demographic and pain surveys including CSI-9, PCS, and Likert pain score (scale 0-10). Participants were randomized to either receive a standard prescription of ten 5 mg tablets oxycodone provided preoperatively (standard) or opioid prescription provided only upon patient request postoperatively (restricted). 82 participants underwent isolated MUS placement and met inclusion criteria; 40 were randomized to the standard arm and 42 to the restricted group Fig. 1). The randomization allocation sequence was generated by the statistician using permuted blocks of random size 2, 4, and 6 with a 1:1 allocation ratio to standard or restricted regimen. The randomization sequence was then uploaded directly into REDCap [3]. Randomization was performed by the study team surgeon using the REDCap randomization module on the day of surgery. Neither providers, nor outcome observers, nor study participants were masked with regards to allocation after inclusion. Participants all received detailed, standardized instruction on non-opioid pain control methods postoperatively, including use of nonsteroidal anti-inflammatories, acetaminophen and heat/ice.

After MUS, subjects completed a daily diary for 1 week, i.e., postoperative day (POD) 0 through 7, to determine average daily pain score, opioid use and amount utilized, other forms of pain management, satisfaction with pain control, perception of the amount of opioid prescribed, and need to return to care for pain management. Diaries were collected electronically via REDCap or on a paper log if no internet access. Patients unable to complete their diary in REDCap received a structured phone call at POD #1 and 7 to collect paper diary information. All participants were also asked to return their paper diary (if applicable) at a postoperative visit. The online Prescription Drug Monitoring Program (PDMP) was queried for all patients to determine if prescriptions for opioids were filled during the postoperative period.

The primary outcome for this non-inferiority study was postoperative day 1 pain at the end of the day based on a Likert scale ranging from 0-10, where higher scores indicate worse pain. Secondary outcomes included whether subject filled an opioid prescription (indicated by the online PDMP), opioid use (yes/no), satisfaction with pain control (on a scale of 1= “much worse” to 5= “much better” than expected), and how subjects felt about the amount of opioid prescribed (on a scale of 1=“prescribed far more” to 3=“prescribed the right amount” to 5=“prescribed far less” opioid than needed). Opioid use was determined using the daily POD0-POD7 diaries and defined as using at least 1 oxycodone 5mg tablet. An average score per participant was calculated for the 5-point Likert scale questions regarding satisfaction and amount of opioid prescribed, which were both assessed on the daily diaries. A psychometric survey and clinical/demographic factors associated with opioid use was an additional secondary outcome.

Using a margin of non-inferiority of 2 points on the Likert pain scale (previously described as a clinically significant difference between the level of pain experienced by vaginal surgery treatment groups) [5] for this two-arm study and standard deviation of 3 points, a sample size of 37 per arm was calculated to provide 81% statistical power to detect non-inferiority between the two arms using a one-sided test having a significance level of 0.025. Taking into consideration the potential for a 10% loss of information for the primary outcome (e.g., drop-out), the target sample size for the study was 84 participants.

Data was exported from REDCap into an Excel document. Analyses were performed using SAS Software, version 9.4 [4]. A one-sided, two-sample t-test was used to test the non-inferiority hypothesis where the margin of non-inferiority was 2 points on the Likert pain scale. Two-sample t-tests were used to compare continuous variables, including area under the curve (AUC) pain scores, average satisfaction scores and average rating of the amount of opioid prescribed. Chi-square tests, or Fisher's exact tests if expected counts were small, were used to compare categorical variables, including whether a prescription was filled and whether opioids were used. Additionally, a linear mixed-model was fit, and contrasts constructed, to compare the two arms at each post-operative day with respect to pain scores.

Exploratory analyses were performed to investigate predictors of postoperative pain score and opioid use. A multiple binary logistic regression model was used to assess the association of baseline factors (PCS score, CSI-9 score, PACU opioid use, baseline pain score, and randomization assignment) with postoperative opioid use. A multiple linear regression model assessed the association of these same baseline factors with postoperative pain score.

## Ethics Statements

Approval was obtained from the Pennsylvania State University College of Medicine Institutional Review Board (STUDY13951) and the study was registered at ClinicalTrials.gov (NCT04277975). Informed consent was obtained from all participants prior to participation.

## CRediT Author Statement

**Brianne Morgan:** Investigation, Writing; **Jaime B. Long:** Conceptualization, Funding acquisition, Investigation, Writing; **Sarah Boyd:** Conceptualization, Investigation; **Matthew Davies:** Conceptualization, Investigation; **Allen Kunselman:** Conceptualization, Data curation, Formal analysis, Writing; **Christy Stetter:** Conceptualization, Data curation, Formal analysis, Writing; **Michael Andreae:** Conceptualization, Investigation, Writing.

## Declaration of Competing Interest

The authors declare that they have no known competing financial interests or personal relationships that could have appeared to influence the work reported in this paper.

## Data Availability

Data in Brief of: Liberal vs. Restricted Opioid Prescribing Following Midurethral Sling (Original data) (Mendeley Data).

## Appendix Supplementary materials

Image, application 1

## References

[1] Long J.B., Morgan B.M., Boyd S.S., Davies M.F., Kunselman A.R., Stetter C.M., Andreae M.H. (2022). A randomized trial of standard vs restricted opioid prescribing following midurethral sling. Am. J. Obstet. Gynecol..

[2] Morgan B., Long J., Boyd S., Davies M., Kunselman A., Stetter C., Andreae M. (2023). Data in Brief of: Liberal vs. Restricted Opioid Prescribing Following Midurethral Sling. Mendeley Data.

[3] Harris P.A., Taylor R., Thielke R. (2009). Research electronic data capture (REDCap) – a metadata-driven methodology and workflow process for providing translational research informatics support. J. Biomed. Inform..

[4] SAS Intitute Inc 2013. SAS/ACCESS 9.4 Interface to ADABAS: Reference. Cary, NC: SAS Institute Inc.

[5] Feroz R.T., Boyd S.S., Schaefer E.W., Swailes A.L., Long J.B. (2021). Postoperative opioid filling patterns in women undergoing midurethral sling placement. Female Pelvic Med. Reconstr. Surg..

