# Supplementary material for: Liberal vs. restricted opioid prescribing following midurethral sling dataset
Source: Data Brief. 2023 Apr 12;48:109144. doi: 10.1016/j.dib.2023.109144 (PMC10293997; doi:10.1016/j.dib.2023.109144)
Supplement: Supplementary file 1 [file mmc1.docx]

**Supplemental Information 1: Postoperative Diary**

| Date: | Average daily pain score  (Rate your pain 0-10, with 0 for no pain and 10 for worst pain imaginable) | Number of tablets of prescribed opioid tablets used | Other medications used for pain  (ibuprofen, acetaminophen) | Other methods of pain control used (heat, ice, pressure) | Satisfaction with pain control  (Rate 1-5 for: much worse (1), worse (2), same (3), better (4) or much better (5) than expected) | Do you have the right amount of prescribed opioid to manage your pain? (Rate 1-5 for: much more than needed (1), more than needed (2), right amount (3), less than needed (4), far less than needed (5)) | Have you required a trip to return to the hospital, clinic, or urgent care for unmet pain needs?  (Y/N) |
| --- | --- | --- | --- | --- | --- | --- | --- |
| Day of surgery: ______ |  |  |  |  |  |  |  |
| Post-op day #1: ______ |  |  |  |  |  |  |  |
| Post-op day #2: ______ |  |  |  |  |  |  |  |
| Post-op day #3: ______ |  |  |  |  |  |  |  |
| Post-op day #4: ______ |  |  |  |  |  |  |  |
| Post-op day #5: ______ |  |  |  |  |  |  |  |
| Post-op day #6: ______ |  |  |  |  |  |  |  |
| Post-op day #7: ______ |  |  |  |  |  |  |  |

Please record your average pain score at end of day prior to going to bed, with 0 indicating “no pain” and 10 indicating “pain as bad as you can imagine”

**Supplemental Information 2: CSI-9 Survey**

Please select “never”, “rarely”, “sometimes”, “often”, or “always” as the best response to the right of each statement

- I feel tired and unrefreshed when I wake from sleeping
- My muscles feel stiff and achy
- I feel pain all over my body
- I have headaches
- I do not sleep well
- I have difficulty concentrating
- Stress makes my physical symptoms get worse
- I have tension in my neck and shoulders
- I have difficulty remembering things

**Supplemental Information 3: PCS Survey**

**Everyone experiences painful situations at some point in their lives. Such experiences may include headaches, tooth pain, joint or muscle pain. People are often exposed to situations that may cause pain such as illness, injury, dental procedures or surgery.

We are interested in the types of thoughts and feelings that you have when you are in pain. Listed below are thirteen statements describing different thoughts and feelings that may be associated with pain. Using the following scale, please indicate the degree to which you have these thoughts and feelings when you are experiencing pain.

0 - not at all 1 - to a slight degree 2 - to a moderate degree 3 - to a great degree 4 - all the time**

- I worry all the time about whether the pain will end
- I feel I can’t go on
- It’s terrible and I think it’s never going to get better
- It’s awful and I feel that it overwhelms me
- I feel I can’t stand it anymore
- I become afraid that the pain will get worse
- I keep thinking of other painful events
- I anxiously want the pain to go away
- I can’t seem to keep it out of my mind
- I keep thinking about how much it hurts
- I keep thinking about how badly I want the pain to stop
- There’s nothing I can do to reduce the intensity of the pain
- I wonder whether something serious may happen
